# Respiratory status determines the effect of emodin on cell viability

**DOI:** 10.18632/oncotarget.16396

**Published:** 2017-03-21

**Authors:** Verónica I Dumit, Ralf M Zerbes, Stephanie Kaeser-Pebernard, Michal Rackiewicz, Mona T Wall, Christine Gretzmeier, Victoria Küttner, Martin van der Laan, Ralf J Braun, Jörn Dengjel

**Affiliations:** ^1^ Freiburg Institute for Advanced Studies FRIAS, University of Freiburg, Freiburg, Germany; ^2^ Center for Biological Systems Analysis ZBSA, Freiburg, Germany; ^3^ Department of Dermatology, Medical Center, University of Freiburg, Freiburg, Germany; ^4^ Core Facility Proteomics, ZBSA, University of Freiburg, Freiburg, Germany; ^5^ Institute of Biochemistry and Molecular Biology, ZBMZ, Faculty of Medicine, University of Freiburg, Freiburg, Germany; ^6^ Faculty of Biology, University of Freiburg, Freiburg, Germany; ^7^ BIOSS Centre for Biological Signalling Studies, University of Freiburg, Freiburg, Germany; ^8^ Medical Biochemistry and Molecular Biology, Center for Molecular Signaling, PZMS, Saarland University, Homburg, Germany; ^9^ Institute of Cell Biology, University of Bayreuth, Bayreuth, Germany; ^10^ Department of Biology, University of Fribourg, Fribourg, Switzerland

**Keywords:** chemoproteomics, reactive oxygen species, mitochondria, anthraquinone, complex I

## Abstract

The anthraquinone emodin has been shown to have antineoplastic properties and a wealth of unconnected effects have been linked to its use, most of which are likely secondary outcomes of the drug treatment. The primary activity of emodin on cells has remained unknown. In the present study we demonstrate dramatic and extensive effects of emodin on the redox state of cells and on mitochondrial homeostasis, irrespectively of the cell type and organism, ranging from the yeast *Saccharomyces cerevisiae* to human cell lines and primary cells. Emodin binds to redox-active enzymes and its effectiveness depends on the oxidative and respiratory status of cells. We show that cells with efficient respiratory metabolism are less susceptible to emodin, whereas cells under glycolytic metabolism are more vulnerable to the compound. Our findings indicate that emodin acts in a similar way as known uncouplers of the mitochondrial electron transport chain and causes oxidative stress that particularly disturbs cancer cells.

## INTRODUCTION

Emodin (6-methyl-1,3,8-trihydroxyanthraquinone) is a naturally occurring anthraquinone present in a wide variety of plants, particularly in roots and barks [1]. Diverse beneficial properties of emodin for human health have been described, such as anti-bacterial [2], anti-fungal [3], and anti-viral effects [4]. It is also suggested for treatment of diabetes [5] and cancer [6, 7]. As antineoplastic agent emodin has been shown to affect many different tumor cell lines inhibiting growth of leukemia, breast, colon, and lung carcinoma cells, amongst others [7]. It is well accepted that cancer cells undergo apoptosis as response to emodin [6]. Yet there is a lack of consensus on the compound's molecular targets and mechanisms. The wealth of controversial reports on emodin is most likely evidence for extensive secondary effects caused by its use rather than a clear explanation of the direct cellular consequences of emodin treatment.

One of the main characteristics of cancer cells compared to healthy ones is an extensive prooxidative state that can lead to intrinsic oxidative stress [8]. Elevated reactive oxygen species (ROS) levels in cancer cells are mainly due to their accelerated metabolism and mitochondrial malfunction [9]. The major site of ROS generation is the mitochondrial respiratory chain, where erroneous single electron reductions of oxygen at different redox centers (electron leakage) lead to ROS production [10]. The high ROS content in cancer cells renders them more susceptible to oxidative stress–induced cell death [9]. Moreover, normal and cancer cells differ in their energy metabolism, a phenomenon referred to as the Warburg effect [11]: compared to healthy cells, most cancer cells rely on anaerobic glycolysis rather than on mitochondrial oxidative phosphorylation to generate ATP and precursor metabolites needed for cellular homeostasis and proliferation [12].

This work originates from the observation that emodin affects cancer cells in culture while healthy cells are apparently resistant to its effects. To unravel the molecular mechanism of the compound, we performed targeted and global proteomics analyses of healthy and cancer cells treated with emodin [13]. We characterize its binding partners and delineate its differential influence on cancer and healthy cells. Considering the different metabolic requirements of cancer and healthy cells, we incorporated a yeast model for respiratory-to-glycolytic metabolic switches allowing us to generalize emodin's effects in eukaryotic cells. This comprehensive approach allowed us to analyze the influence of metabolic stages on the effects of emodin and supports the hypothesis that emodin's differential effects in cancer and healthy cells depend on their differential mitochondrial fitness.

## RESULTS

### Emodin negatively affects cell proliferation

**Figure 1:**
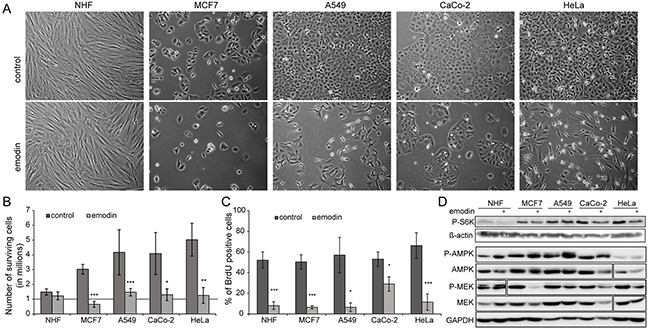
Effect of emodin on healthy and cancer cells Evaluated cells are normal human fibroblasts (NHF) and four cancer cell lines: MCF7, A549, CaCo-2 and HeLa. **(A)** Phenotype of cells treated with 70 μM emodin after 48 h compared to control treatment. **(B)** Relative number of cells after two days of treatment. Initial number of cells is one million and it is highlighted by a solid grey line. **(C)** Quantification of BrdU assay of cells treated for 24 h with 70 μM emodin compared to control conditions to determine percentage of proliferating cells. **(D)** Western blots to detect levels of phospho-ribosomal protein S6 kinase (P-S6K, Thr389) in control and emodin treated cells. Actin was used as a loading control. For phospho-AMPKα (P-AMPK, Thr172) and phospho-MEK (P-MEK, Ser217/221) western blots, the respective non-modified proteins and GAPDH were used as loading control. The P-MEK blot of NHF shows a higher exposure.

Emodin has a detrimental effect on cancer cells in culture, while healthy cells apparently remain unaffected by the compound. In this study, we evaluated the response of primary normal human fibroblasts from skin (NHF), normal human keratinocytes (NHK), and of MCF7, A549, CaCo-2 and HeLa cells, which are cancer cell lines isolated from breast, lung, colon and cervix carcinomas, respectively, to emodin. Figure 1A and Supplementary Figure 1 show the phenotypes of above mentioned cells after 48 h of 70 μM emodin treatment versus control conditions. Notably, numbers of NHF and NHK were not affected by the treatment, while numbers of cancer cells were significantly reduced to 20-30% for all cancer cell lines (Figure 1B, Supplementary Figure 1B). Reduced cancer cell numbers could be due to increased cell death and/or decreased cell proliferation. As the numbers of cells after 48 h of emodin treatment were not less than the respective starting numbers, except in the case of MCF7 cells, we reasoned that differences in cell proliferation were the main cause for the observed phenotypes. To discriminate between increased cell death and decreased cell proliferation we analyzed apoptosis and proliferation directly. We only found minor signs of apoptosis (data not shown), however, cell proliferation was significantly affected by emodin in both healthy and cancer cells as analyzed by 5-bromo-2’-deoxyuridine (BrdU) incorporation assays (Figure 1C, Supplementary Figure 2). Thus, in the performed experiments emodin appeared to act primarily cytostatic having limited cytotoxic effects. Due to increased doubling times healthy cells appeared less affected than cancer cells.

To study the molecular basis of decreased cell proliferation by emodin treatment we analyzed signaling pathways known to play a role in cell proliferation and growth. Analysis of mechanistic target of rapamycin (MTOR) kinase activity by measuring the levels of phosphorylation of its direct target ribosomal protein S6 kinase revealed a perturbation by emodin, in correlation with decreased cell proliferation and protein synthesis (Figure 1D, Supplementary Figure 1C). It is evident that under control conditions the levels of phosphorylation of ribosomal protein S6 kinase were higher in cancer cells than in healthy cells. Upon emodin treatment, phosphorylation levels decreased in all cells, A549 cells responding the least. In agreement with a decrease of cell proliferation also mitogen-activated protein kinase (MAPK) signaling was negatively affected in cancer cells by emodin treatment as indicated by a decreased activating phosphorylation of MAPK/ERK kinase (MEK) 1 and 2. In contrast AMP-activated protein kinase (AMPK) activity appeared to increase upon emodin treatment indicating increased cell stress (Figure 1D, Supplementary Figure 1C). Thus, emodin treatment led to a cell line independent decrease of cell growth- and proliferation-stimulating signals, increasing cell stress.

### Emodin binds to redox-active enzymes increasing oxidative stress

**Figure 2:**
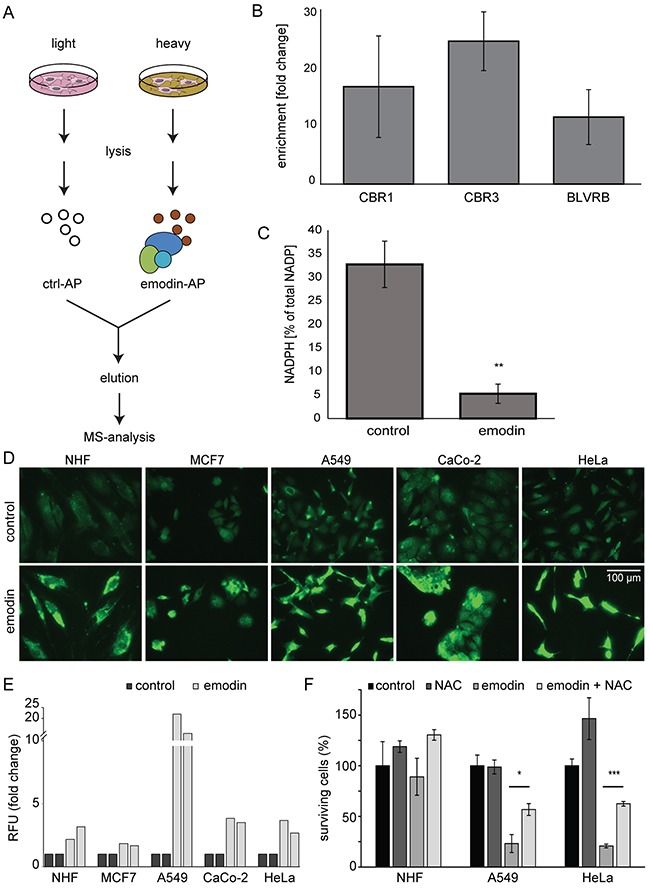
Analysis of direct emodin target proteins **(A)** SILAC-based chemoproteomics approach to determine emodin target proteins. AP: affinity purification **(B)** Enrichment of NADPH binding proteins carbonyl reductase 1 and 3 (CBR1, CBR3) and flavin reductase (BLVR) by affinity purification with emodin-coated beads. The mean from three biological replicates is shown. Error bars indicate SEM. **(C)** Percentage of NADPH relative to total NADP^+^-NADPH pool in control and emodin treated cells. Statistics: Unpaired two-tailed Student's t-test. **: p < 0.01 and n=3. Error bars indicate SD. **(D)** Fluorescence microscopy demonstrates increased levels of ROS in all cells after emodin treatment. **(E)** Fold changes in relative fluorescence units (RFU, Ex/Em = 530/580 nm) normalized to cell numbers and control conditions indicate an increase in H_2_O_2_ levels upon emodin treatment. Shown are results from two biological replicates for each cell type. **(F)** Relative number of surviving cells after emodin treatment and the influence of NAC. Mean values of three measurements are shown. Error bars indicate SD. *: p < 0.05; ***: p < 0.001.

To identify direct protein targets of emodin we performed affinity purification-based chemoproteomics experiments. Stable isotope labeling by amino acids in cell culture (SILAC)-based quantitative mass spectrometry (MS) of CaCo-2 cells was employed to identify proteins binding to emodin-coated sepharose beads compared to ethanolamine-blocked control beads (Figure 2A, Supplementary Table 1) [14]. In three biological replicates we identified ten proteins as enriched exhibiting significant changes in minimally two out of the three experiments. Interestingly, three of these have a nicotinamide adenine dinucleotide phosphate (NADPH) binding domain as intrinsic characteristic: carbonyl reductases 1 and 3 and flavin reductase, all being NADPH-dependent oxido-reductases (Figure 2B). Thus emodin, which has a central quinone group, bound to redox-active enzymes, possibly interfering with their function. To analyze if emodin treatment has a global effect on NADPH levels we quantified NADPH by a colorimetric assay. Indeed, emodin treatment led to a significant decrease of NADPH in the total pool of NADP^+^ and NADPH (Figure 2C).

Next to its important role as cofactor in anabolic processes, NADPH is vital for other reductive processes: NADPH is cofactor of glutathione reductase which recycles glutathione, an important cellular antioxidant. Therefore, decreased NADPH levels may indicate increased oxidative stress in emodin treated cells. To analyze ROS levels we performed fluorescence microscopy using a fluorescent ROS marker and found that emodin treatment led to increased ROS levels (Figure 2D). We also directly analyzed H_2_O_2_ levels and detected an increase in all cells treated with emodin (Figure 2E). To determine if ROS play a role in the detrimental effects of emodin on cancer cells, we compared the effects of emodin alone or in combination with the ROS scavenger N-acetyl-L-cysteine (NAC). Additional antioxidant treatment partially protected cells and led to an increase in the number of cells after emodin treatment, especially in the case of cancer cell lines (Figure 2F). Taken together, emodin directly bound to redox-active enzymes and interfered with the cellular NADPH pool. Reduced NADPH was linked to higher ROS levels observed in emodin-treated cells. ROS scavengers promoted survival of cancer cells in the presence of emodin, whereas the influence on healthy cells was limited.

### Global proteomics analyses reveal an effect of emodin on mitochondria

**Figure 3:**
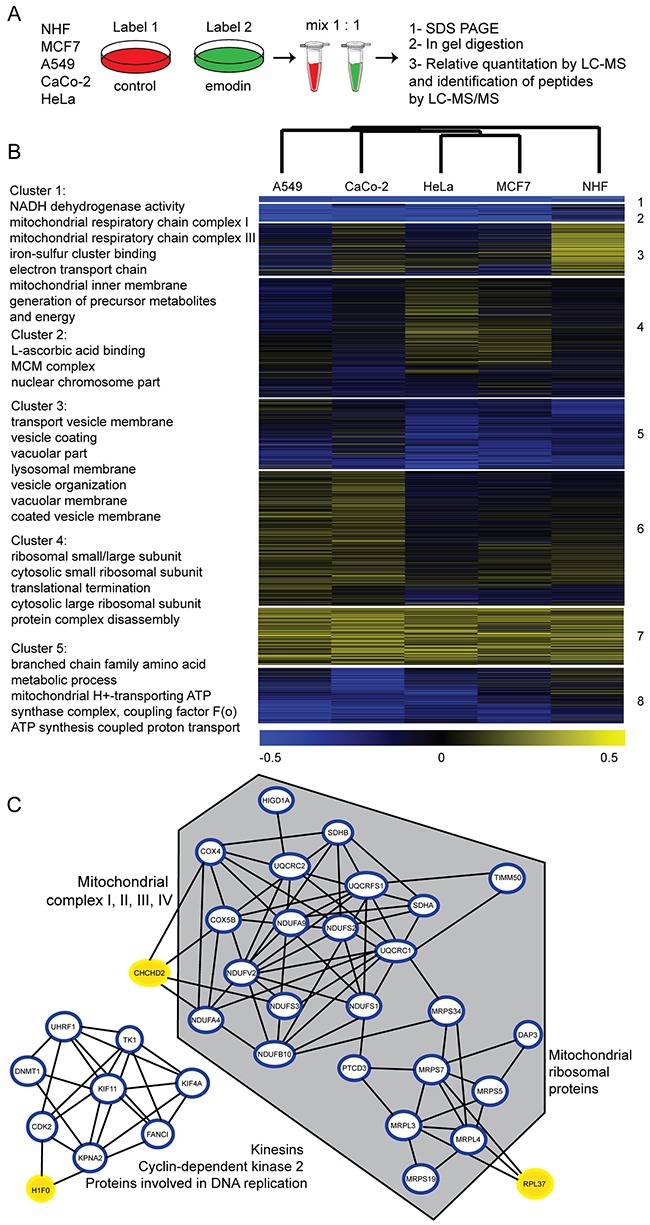
Expression proteomic analysis of cells treated with emodin **(A)** Chemoproteomics workflow. Cells were treated for 24 h by 70 μM emodin. **(B)** Heat map of log_2_ transformed SILAC ratios of cells treated with emodin compared to respective controls (two biological replicates each). k-means clusters and shared GO terms of each cluster are described on the left (4 fold enrichment, p-value < 0.05). **(C)** Interaction network of significantly altered proteins in at least two cell types. Grey hexagon indicates mitochondrial proteins, blue circled proteins are significantly down-regulated and yellow-marked proteins are significantly up-regulated under emodin treatment (p<0.05).

To gain comprehensive insight into the physiological consequences of emodin treatment and underlying cellular responses leading to the observed phenotypes, we chose an unbiased and global chemoproteomics approach [15]. We analyzed whole cell lysates of five cell types included in this study by SILAC-based quantitative expression proteomics experiments. We fully labeled cells with isotopically labeled amino acids, and subjected them to control or 24 h emodin treatment. Whole cell lysates of each cell type corresponding to both control and treatment conditions were mixed in a 1:1 protein ratio. Proteins in each mix were analyzed by gel-liquid chromatography (LC)-MS/MS (Figure 3A). Biological replicates were performed (Supplementary Figure 3) and respective data were merged. In total, we detected 4,191 proteins of which we could quantify 3,761 (Supplementary Table 2). Of these 2,008 proteins were identified in all five cell types (Supplementary Table 3).

Principal component analysis revealed that healthy cells reacted profoundly different to emodin compared to cancer cells (Supplementary Figure 4). To further characterize these differences, the data were subjected to hierarchical clustering to study the relationship between samples, and k-means clustering to group proteins (Figure 3B). Proteins separated into eight k-means clusters, the response of NHF being clearly distinct from all cancer cell lines. Proteins in each k-means cluster were tested on enriched gene ontology (GO) terms [16] (p-value < 0.05). Cluster 3 contains vesicular and lysosomal proteins which were more abundant in NHF compared to all cancer cell lines. Cluster 1 includes proteins which were down-regulated in all evaluated cells. Interestingly, the majority of these proteins belong to the mitochondrial respiratory chain. Particularly down-regulated were complex I protein components (NDUF and MT-ND proteins) upon 24 h of emodin treatment. Other respiratory chain complexes were also affected (complex II, III, IV), but to a lesser extent. Figure 3C displays known or predicted interactions of those proteins which were significantly down-regulated in at least two of the evaluated cell types upon 24 h of emodin treatment as assessed by STRING DB analysis (p<0.05) [17]. Especially the above mentioned respiratory chain subunits and also mitochondrial ribosomal proteins, in contrast to cytosolic ribosomal proteins, both of the large and small subunit, were reduced by half after treatment (Figure 3C, Supplementary Table 3). Interestingly, the oxygen sensor CHCHD2 which was shown to activate expression of genes containing oxygen responsive elements in their promoters under hypoxic conditions was upregulated [18], indicating a general perturbation of oxidative metabolism in emodin-treated cells.

Emodin seemed to have only minor effects on the abundance of other mitochondrial proteins. For example, components of the pyruvate dehydrogenase complex (PDH), and of the citric acid cycle (aconitate hydratase, malate dehydrogenase and fumarate hydratase) appeared unaffected. Levels of marker proteins of the mitochondrial outer and inner membrane were unaltered, as well. Classical effectors of mitochondrion-associated apoptosis (cytochrome *c* and apoptosis-inducing factor) were mostly unaffected (Supplementary Figure 5). Nonetheless, our measurements cannot precise the cellular location of these proteins nor distinguish between their pro- or active-forms.

Figure 3C also shows a cluster of interacting cytosolic proteins that are known to be involved in cell proliferation and cell cycle, which were also significantly downregulated by emodin treatment. This result is in agreement with the observed decrease in proliferation rates. Taken together, emodin affected the proteome of healthy cells differently compared to those of cancer cells. Our analyses suggest next to redox-active enzymes mitochondria as its prime site of action.

### Emodin treatment decreases complex I levels and induces mitochondrial fragmentation

**Figure 4:**
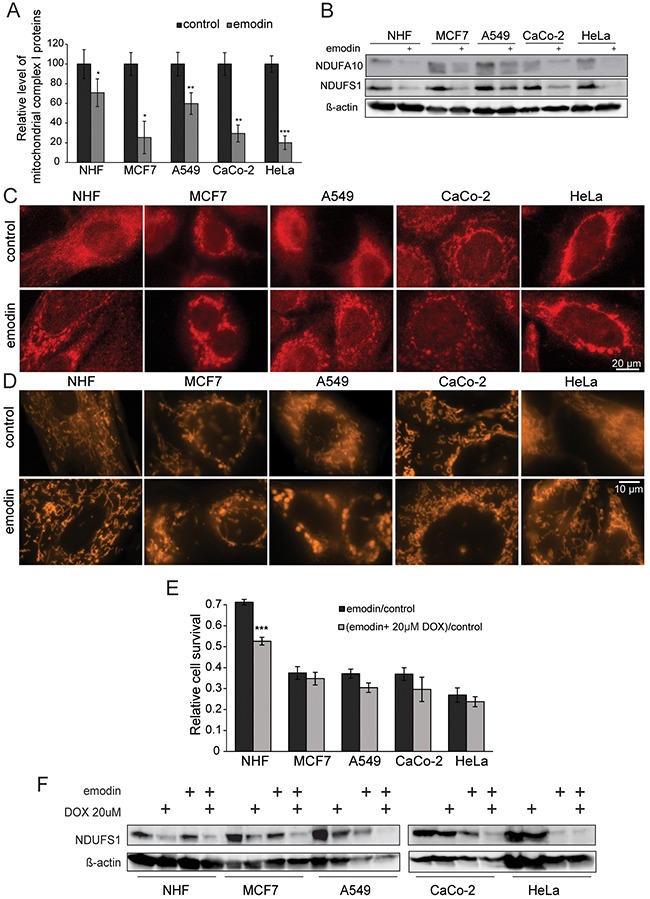
Emodin leads to mitochondrial fragmentation and ROS generation **(A)** Average levels of all mitochondrial proteins of complex I of the electron transport chain as detected by SILAC-based MS (mean values of four different complex I proteins). **(B)** Western-blots show the decrease of NDUFA10 and NDUFS1 of mitochondrial complex I in all evaluated cells. Actin served as a loading control. **(C)** NDUFS1 staining in fixed cells exhibits fragmentation of the mitochondrial network. **(D)** MitoTracker staining of live cells confirms mitochondrial network fragmentation observed in panel **(C)**. **(E)** DOX pretreatment of cells renders healthy cells more susceptible to emodin, while cancer cells are not significantly affected (mean values of three independent experiments). **(F)** Western blot anti-NDUFS1, a nuclear encoded protein of respiratory complex I, under emodin treatment after pretreatment with DOX. Actin was used as a loading control. Error bars: standard deviation. Unpaired two-tailed Student's t-test. *: p < 0.05, **: p < 0.01, ***: p < 0.001.

As detected by MS, levels of all mitochondrial complex I proteins decreased after emodin treatment in all cells analyzed. However, emodin affected the levels of complex I proteins to a lesser extent in healthy fibroblasts than in cancer cells (Figure 4A). Western blot analyses against the nuclear encoded complex I proteins NDUFA10 and NDUFS1 were in agreement with MS results (Figure 4B). To study morphological effects of emodin treatment we performed immunofluorescence microscopy employing an anti-NDUFS1 antibody with PFA-fixed cells. After emodin treatment mitochondria appeared fragmented (Figure 4C), which was also evident from MitoTracker staining of live cells (Figure 4D). Both staining exhibit swollen mitochondria, clearly demonstrating mitochondrial stress caused by emodin. Mitochondrial network fragmentation upon emodin treatment was in agreement with MS results, which also showed decreased levels of the mitochondrial fusion protein OPA1 and of the protease YME1L1 that is involved in proteolytic processing of OPA1 [19] after emodin treatment (Supplementary Figure 6).

Compared to healthy cells, mitochondria in cancer cells function less efficiently leading to higher basal ROS levels in cancer cells (Supplementary Figure 7). To determine the role of mitochondrial fitness in the cellular response to emodin, we used doxycyclin (DOX), an antibiotic known to affect mitochondria by binding to the 28S mitochondrial ribosome subunit [20–22]. We treated cells prior to emodin treatment with DOX and evaluated their response. Notably, DOX pretreatment of cells rendered healthy cells more sensitive to emodin, while cancer cells were not significantly affected (Figure 4E). By western blot we show that DOX diminished levels of NDUFS1, which were even more decreased by emodin (Figure 4F). These experiments clearly indicate that good mitochondrial fitness is a prerequisite to overcome the effects of emodin treatment.

### High respiratory capacities protect from ROS production and emodin sensitivity

**Figure 5:**
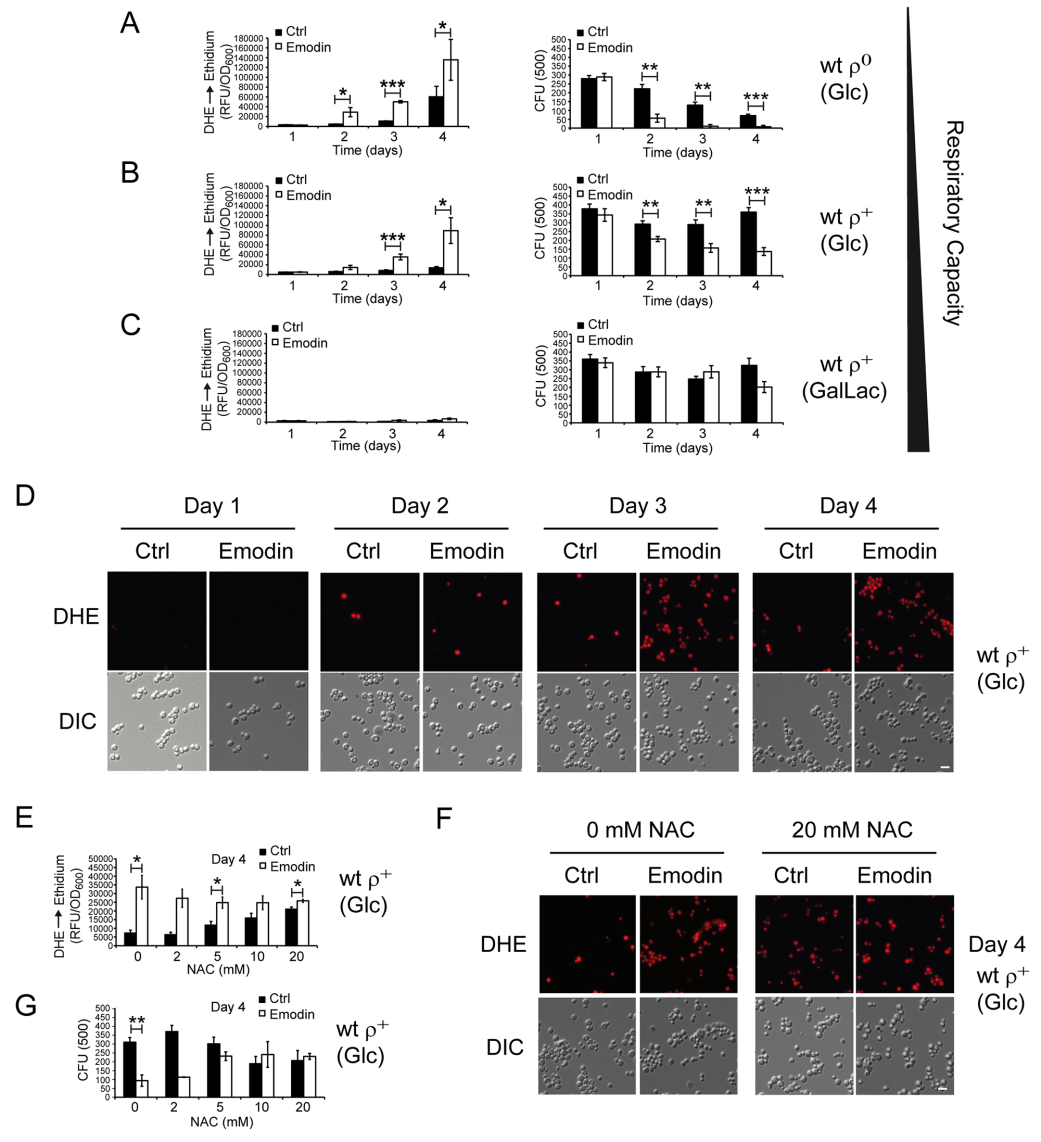
Yeast cells with low respiratory capacities demonstrate critical ROS accumulation upon emodin treatment culminating in loss of cell survival **(A-C)** Wild-type ρ^+^ strain with intact mtDNA and ρ^0^ strain lacking mtDNA were tested for cytotoxic effects of emodin. ρ^0^ strain was grown on medium with glucose (Glc) as carbon source (fermentation only) **(A)**, and wild-type ρ^+^ strain was either grown on media with glucose (Glc) as carbon source (respiration is possible but repressed by glucose) **(B)** or on media with galactose/lactate (Gal/Lac) as carbon source (high respiratory rates) **(C)**. Yeast cells were tested for ROS levels (DHE to ethidium conversion; relative fluorescence unit (RFU) *vs*. cell mass (OD_600_); left panels) and for cell survival (colony forming unit, CFU; right panels) without and with 50 μM emodin, respectively, at days 1, 2, 3, and 4 after begin of treatment. Data shown here a mean values of seven experiments. **(D)** Representative micrographs of ROS accumulation as quantified in **(B)**. **(E-G)** Wild-type ρ^+^ strain was grown on medium with glucose as carbon source for four days without and with 50 μM emodin in the presence of increasing concentrations of NAC. ROS accumulation **(E, F)** and cell survival **(G)** at the different conditions. Data shown are mean values of three independent experiments. Error bars: standard deviation. Statistics: Unpaired two-tailed Student's t-test. *: p < 0.05, **: p < 0.01. Scale bars in (D+F): 10 μm.

With the aim of further studying the sensitivity of cells with different respiratory capacities to emodin, we employed the yeast *Saccharomyces cerevisiae*. Yeast cells can readily switch between fermentation and oxidative phosphorylation, and the respiratory capacities of yeast cultures can easily be modulated by genetic intervention or by changing the main carbon sources in the growth media [23]. Yeast cells lacking mitochondrial DNA (ρ^0^ cells) are incapable to perform respiration, due to the lack of mitochondrial DNA-encoded essential subunits of the respiratory chain, and therefore solely depend on fermentation. ρ^0^ cells use ATP to maintain the basal mitochondrial membrane potential, which is a prerequisite for essential mitochondrial functions, such as Fe/S cluster biosynthesis [24] and protein import [25]. Consequently, ρ^0^ cells are still able to undergo mitochondrion-dependent cell death [26], and to produce high levels of ROS [27, 28]. In contrast, yeast cells with intact mitochondrial DNA (ρ^+^ cells) can switch their metabolism between fermentation and respiration dependent on the availability of the main carbon sources. In medium with glucose (Glc) as carbon source yeast cells undergo fermentation, mainly because of catabolite repression of respiratory enzymes by the presence of glucose [29]. In contrast, GalLac medium contains galactose and lactate as carbon sources and stimulates yeast cells to respire. In order to analyze emodin-triggered effects in yeast cells with a broad range of respiratory capacities, we treated ρ^0^ cells grown on glucose containing media (Glc) (no respiratory capacity), ρ^+^ cells grown on glucose containing media (Glc) (respiration is repressed by glucose but basal respiratory capacity is maintained), and ρ^+^ cells grown on galactose/lactate (GalLac)-containing media (high respiratory capacities). Yeast cultures treated for one to four days with emodin were evaluated for levels of oxidative stress, which can be detected by the intracellular conversion of the ROS-sensitive stain dihydroethidium (DHE) to fluorescent ethidium [30]. This approach was paralleled by determining the proportion of surviving cells (colony forming units, CFU) on nutrient-containing solid medium. Depending on the respiratory capacity, yeast cells show different levels of oxidative stress due to emodin treatment and different survival rates (Figure 5A–5D). The higher the respiratory capacity of the yeast, the lower the levels of oxidative stress triggered by emodin treatment, possibly due to a densely packed and highly efficient respiratory chain, and due to a highly efficient anti-ROS defense system. The survival of yeast cultures as well as the resistance against emodin were higher in respiring cells. Remarkably, a functional mitochondrial respiratory chain is not necessary for the cytotoxic effects of emodin, because emodin reliably kills cells lacking a functional respiratory chain (ρ^0^ strain under Glc conditions). A highly active and efficient mitochondrial respiratory chain protects from emodin (ρ^+^ strain, GalLac), whereas cells with an intact respiratory chain but that primarily undergo fermentation (ρ^+^ strain, Glc) are highly sensitive to emodin. Thus, the respiratory capacity of yeast cells determines the cytotoxicity of emodin.

Next, we asked whether ROS production is a cause for or a consequence of emodin-triggered cell death in yeast. Therefore, we incubated emodin-treated yeast cells (ρ^+^ strain, Glc) with different concentrations of the antioxidant NAC, and measured the levels of ROS and yeast cell survival. We observed that increasing the concentration of NAC reduced the levels of oxidative stress in cells treated with emodin and increased the levels of oxidative stress in control cells (Figure 5E–5F). Consistently, elevated NAC levels were protective for cells treated with emodin and were moderately harmful for control cells (Figure 5G). Thus, emodin demonstrated significantly reduced abilities to boost the levels of oxidative stress and to kill the cells in the presence of NAC. These data suggest that ROS are causatively involved in the cytotoxic effects of emodin in yeast cells.

### Emodin uncouples mitochondria and impairs mitochondrial protein import

**Figure 6:**
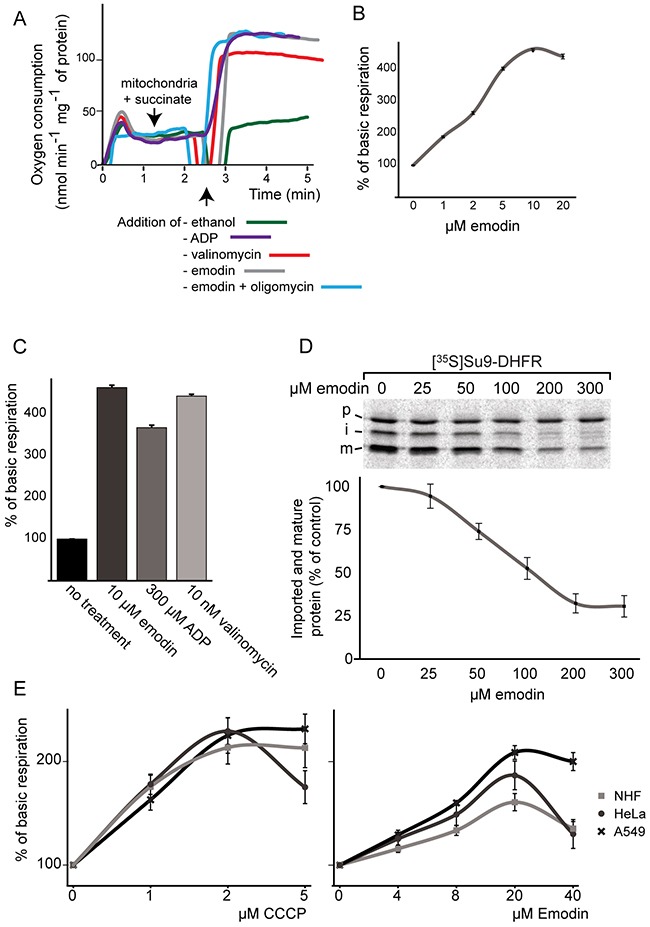
Effect of emodin on oxygen consumption and respiration in mitochondria, healthy and cancer cells **(A, B, C)** Oxygen consumption of isolated yeast mitochondria under emodin treatment. **(D)** Import of an radiolabelled [^35^S]Su9-DHFR, a model protein targeted to the mitochondrial matrix, into isolated yeast mitochondria (p=precursor; i=intermediate; m=mature) in the absence or presence of different emodin concentrations. The lower panel displays quantifications of the mature (m) protein bands in three independent experiments. The signal obtained in the absence of emodin was set to 100%. Error bars represent SEM. **(E)** Comparison of respiration of healthy NHF, A549 and HeLa cells under emodin and CCCP treatment. Error bars represent SEM; n = 3.

To further study the physiological consequences of emodin treatment and outline its effects on mitochondrial biology, we investigated mitochondrial oxygen con-sumption and inner membrane potential (Δψ). Emodin strongly increased oxygen consumption of isolated yeast mitochondria in the presence of respiratory substrates (Figure 6A–6C). Addition of ADP to induce coupled (state III) respiration or valinomycin, an ionophore of the mitochondrial inner membrane, to reveal the maximal respiratory capacity caused a similar increase as the addition of emodin. Additional block of the ATP synthase by oligomycin did not prevent the increase in oxygen consumption by the addition of emodin (Figure 6A). We therefore speculated that emodin may possess an uncoupling activity. To demonstrate the effects of emodin on the mitochondrial membrane potential, we monitored an essential conserved Δψ-dependent function of the mitochondrial inner membrane: import of presequence-carrying precursor proteins into the mitochondrial matrix via the presequence translocase (TIM23 complex). The model precursor protein Su9-DHFR [31] was synthesized in a cell-free transcription/translation system in a radio-labelled form and incubated with isolated yeast mitochondria [32]. As shown in Figure 6D, the import efficiency to the mitochondrial matrix was gradually impaired by higher concentrations of emodin. This finding is in agreement with a reduction of mitochondrial Δψ upon addition of emodin and supports the view that the compound has an uncoupling activity.

It is known that the physiological status of mitochondria in cancer cells differs from that in healthy controls. We evaluated the influence of emodin on the mitochondrial activity of healthy NHF, and as examples for cancer cells on A549 and HeLa cells to characterize the extent to which these cells were affected by the compound (Figure 6E). Interestingly, cancer cells reacted with a significantly stronger increase in oxygen consumption to emodin treatment compared to healthy cells. Moreover, in agreement to emodin's influence on cellular NADPH levels, these measurements suggest that emodin has additional effects on mitochondrial metabolism in cancer cells and does not exclusively act as an uncoupler, since the protonophore carbonyl cyanide m-chlorophenyl hydrazine (CCCP) equally increased the oxygen consumption of both cancer and healthy cells. Importantly, treatment by both valinomycin and – to a lesser extent – CCCP did affect the survival of healthy cells similarly to cancer cells further supporting the notion of additional effects of emodin treatment (Supplementary Figure 8).

## DISCUSSION

Emodin proved to affect mammalian cells in a differential manner, dramatically interfering with cancer cell viability, without noticeable consequences for healthy cells. With the aim of understanding the molecular mechanism by which emodin causes these differential effects, we based our studies on targeted and unbiased MS-based chemoproteomics. We identified emodin binding partners and in a global fashion analyzed proteomic changes of cancer and healthy cells upon emodin treatment. Emodin interferes with the cellular redox system by binding to NADPH-dependent oxidoreductases. In addition, in all tested cells emodin significantly affects levels of mitochondrial proteins, particularly of subunits of the respiratory chain complexes. The clearest effect of emodin is the marked decrease in complex I proteins, which is observed more effectively in cancer cells compared to healthy ones. All other electron transport chain complexes show lower protein levels after emodin treatment, however to a lesser extent than complex I.

The yeast results are particularly interesting since they demonstrate that the respiratory capacity of cells is of critical importance for the cell's susceptibility to emodin. The compound particularly damages cells kept in fermentative growth conditions. Increasing the respiratory capacity of yeast cells protects them from the lethal effect of emodin. Along these lines, it seems intuitive to expect ρ^0^ yeast cells to be more resistant to emodin treatment, since they do not rely on mitochondria to generate ATP. However, mitochondria perform key cellular functions other than respiration. Also ρ^0^ cells, which cannot respire, do have a (reduced) mitochondrial membrane potential, largely generated by electrogenic transport processes, like the uptake of ATP from the cytosol in exchange with ADP generated in the mitochondrial matrix by the uncoupling hydrolytic activity of the F_1_-ATPase. Mitochondria are functionally important in these cells for other metabolic processes including Fe/S cluster assembly [24]. It is plausible that interference of emodin with the low membrane potential of ρ^0^ cells through its uncoupling activity is immediately adverse to their survival.

Although the Warburg effect is as yet a matter of discussion, it is accepted that cancer cells rely more on glycolysis than on respiration to obtain energy. As seen from the experiments in yeast, emodin specifically affects cells with a strong fermentative metabolism, but spares cells with high respiratory rates. Thus, cells with high respiratory capacity, like healthy cells, are protected from emodin, whereas cells with less respiratory capacity, as cancer cells, are sensitive to emodin. In well agreement, pre-incubation of mammalian cells with DOX, known to affect 28S mitochondrial ribosomal subunits, renders healthy cells more sensitive to emodin treatment, whereas cancer cells are unaffected by the additional DOX treatment. The marked fragmentation of mitochondria and the strong decrease of mitochondrial proteins in treated cells suggest additional consequences on mitochondria other than the decrease in complex I proteins. As observed by MS, the depletion of the mitochondrial fusion protein OPA1 and of the protease YME1L1 correlates to the observed mitochondrial fragmentation upon emodin treatment. Of note, it has been shown that dissipation of Δψ induces enhanced proteolytic cleavage of OPA1 by the protease OMA1 leading to the fragmentation of the mitochondrial network [33–35].

The direct protein targets of emodin proved to be redox-active enzymes. Nevertheless, our data suggest that another critical molecular mechanism of emodin action is the partial uncoupling of mitochondria. As the depletion of the membrane potential occurs immediately after the addition of emodin to isolated mitochondria, judged by analysis of membrane-potential-dependent preprotein import and oxygen consumption rates of mitochondria (Figure 6), this effect is an early event. We conclude that uncoupling is a direct consequence of emodin treatment and unlikely to depend on its interaction with redox-active enzymes. We demonstrated increased ROS levels by fluorimetric assays both in mammalian and yeast cells. The collective term ROS comprises a whole set of reactive molecules ranging from H_2_O_2_, which is also an important signaling molecule, to highly toxic chemicals such as superoxide anions or hydroxyl radicals. We demonstrated increased H_2_O_2_ levels, but cannot rule out that the levels of other ROS are also altered. Of note, respiratory chain-derived ROS are primarily superoxide anions that are subsequently converted to H_2_O_2_ [36]. The uncoupling activity of emodin does not necessarily explain the concomitant increase in ROS production; both effects may be individual, yet additive. A proof of the primary toxic consequences of emodin-induced ROS is the attenuated effect of emodin in the presence of NAC, a ROS scavenger, in all evaluated eukaryotic cells. Increased ROS levels may also contribute to decreased NADPH levels via the glutathione reductase system. The exact nature, sources and consequences of ROS induced by emodin will have to be addressed in future studies.

Altogether, the majority of the published effects of emodin appear to be of secondary nature. This comprehensive study identifies emodin as binding to NADPH-dependent redox-active enzymes and as a mitochondrial uncoupler, decreasing mitochondrial membrane potential. These alterations lead to an increased oxygen consumption and elevated oxidative stress levels causing a decrease in the levels of complex I [37], possibly to avoid further ROS production. Cells with a high respiratory capacity and a better antioxidant state are better prepared to survive the cellular consequences of the treatment, while cells which mostly rely on fermentative energy production, like cancer cells, are more susceptible to emodin treatment.

## MATERIALS AND METHODS

Additional Materials and Methods can be found online in supplementary information.

### Cell culture and SILAC labeling

Cells were cultured in DMEM (high glucose) supplemented with 10% fetal calf serum, penicillin/streptomycin (100 U/mL, 100 μg/mL), and 2 mM l-glutamine. For the labeling, cells were cultured in SILAC-DMEM supplemented with 10% dialyzed fetal calf serum, penicillin/streptomycin (100 U/mL, 100 μg/mL), 2 mM l-glutamine, 42 mg/L l-arginine 13C6-15N4, 73 mg/L l-lysine 13C6-15N2 (Arg10-Lys8), and 82 mg/L proline.

### Protein concentration determination

8 × 10^5^ cells were seeded per 10 cm plate and harvested the next day using a cell scraper. Harvested cells were lysed with 80 μL of SDS lysis buffer, incubated for 20 min with benzonase, and centrifuged at 16.000 rcf for 15 min. Supernatant was used for determination of protein concentration employing the Thermo Scientific Pierce BCA Protein Assay Kit as recommended by the supplier.

### ROS level determination

CellROX® Green Reagent from ThermoFisher Scientific was used as recommended by the manufacturer on live cells.

## SUPPLEMENTARY MATERIALS METHODS, FIGURES AND TABLES








