# Supplementary material for: Respiratory status determines the effect of emodin on cell viability
Source: Oncotarget. 2017 Mar 21;8(23):37478–90. doi: 10.18632/oncotarget.16396 (PMC5514923; doi:10.18632/oncotarget.16396)
Supplement: Supplementary file 1 [file oncotarget-08-37478-s001.pdf]

Respiratory status determines the effect of emodin on cell viability

SUPPLEMENTARY MATERIALS AND METHODS

Western blot

Proteins were resolved by SDS-PAGE, and were transferred onto a polyvinylidene difluoride (semidry blot) or nitrocellulose (wet blot) membranes. Antibodies against p70 S6 kinase (Cell Signalling #9202S), NDUFA10 (C-14)-R (Santa Cruz, sc-107887-R), NDUFS1 (E-20) (Santa Cruz, sc-50132), actin (Santa Cruz, sc-47778;), P-AMPK (Cell Signaling #2535S), AMPK (Cell Signaling #2532S), P-MEK 1,2 (S217/221; Cell Signaling #9121L), MEK 1,2 (Cell Signaling #9122S), GAPDH-HRP (Santa Cruz, sc-25778HRP) were used according to the supplier's manual, followed by appropriate horseradish peroxidase–conjugated secondary antibodies. The enhanced chemiluminescence method was used for detection on a LAS-4000 Mini (GE Healthcare, Freiburg, Germany).

Immunofluorescence staining

For indirect immunofluorescence staining, fibroblasts grown on cover slips were fixed with 4% PFA and blocked with 2% BSA in PBS for 30 min at room temperature, followed by incubation with the primary antibody diluted in 0.2% BSA in PBS for 1 hour at room temperature. After incubation with the secondary antibody in 0.2% BSA in PBS for 1 hour, the samples were embedded in DAPI containing fluorescence mounting medium (ProLong® Gold, Invitrogen, Darmstadt, Germany). The following primary antibodies were used: cleaved caspase-3 (Cell Signalling #9661). Alexa488–conjugated secondary antibodies (Invitrogen, Darmstadt, Germany) were used. Pictures were taken with an IF microscope (Zeiss Axio Imager, Zeiss, Oberkochen, Germany).

MS sample preparation and measurements

Harvested cells were resuspended in lysis buffer (100 mM Tris pH 7.6; 4% SDS supplemented with protease inhibitor cocktail). Benzonase was added before reducing the samples with 1 mM DTT for 5 min at 95°C and alkylated using 5.5 mM iodacetamide for 30 min at 25°C. Protein mixtures were separated by SDS-PAGE (4-12% Bis-Tris mini gradient gel), gel lanes were cut into 10 equal slices, and in-gel digested using trypsin. Resulting peptide mixtures were processed on STAGE tips as described.

Samples analyzed by MS were measured on LTQ Orbitrap XL mass spectrometer (Thermo Fisher Scientific, Bremen, Germany) coupled either to an Agilent 1200 nanoflow-HPLC (Agilent Technologies GmbH, Waldbronn, Germany) or an Eksigent NanoLC-ultra. HPLC-column tips (fused silica) with 75 µm inner diameter were self-packed with Reprosil-Pur 120 ODS-3 to a length of 20 cm. No pre-column was used. Peptides were injected at a flow of 500 nl/min in 92% buffer A (0.5% acetic acid in HPLC gradient grade water) and 2% buffer B (0.5% acetic acid in 80% acetonitrile, 20% water). Separation was achieved by a linear gradient from 10% to 30% of buffer B at a flow rate of 250 nl/min. The mass spectrometer was operated in the data-dependent mode and switched automatically between MS (max. of 1 x 10^6 ions) and MS/MS. Each MS scan was followed by a maximum of five MS/MS scans in the linear ion trap using normalized collision energy of 35% and a target value of 5,000. Parent ions with a charge states of z = 1 and unassigned charge states were excluded from fragmentation. The mass range for MS was m/z = 370 to 2,000. The resolution was set to 60,000. MS parameters were as follows: spray voltage 2.3 kV; no sheath and auxiliary gas flow; ion transfer tube temperature 125°C.

Data analysis

The MS raw data files were uploaded into the MaxQuant software version 1.4.1.2 [1] which performs peak and SILAC-pair detection, generates peak lists of mass error corrected peptides and data base searches. A full length human Uniprot database containing common contaminants, such as keratins and enzymes used for in-gel digestion, was employed, carbamidomethylcysteine was set as fixed modification and methionine oxidation and protein amino-terminal acetylation were set as variable modifications. Double SILAC was chosen as quantitation mode. Three miss cleavages were allowed, enzyme specificity was trypsin/P, and the MS/MS tolerance was set to 0.5 Da. The average mass precision of identified peptides was in general less than 1 ppm after recalibration. Peptide lists were further used by MaxQuant to identify and relatively quantify proteins using the following parameters: peptide, and protein false discovery rates (FDR) were set to 0.01, maximum peptide posterior error probability (PEP) was set to 0.1, minimum peptide length was set to 6, minimum number peptides for identification and quantitation of proteins was set to two of which one must be unique, and identified proteins have been re-quantified. The "match-between-run" option (2 min) was used.

For the generation of the heat map by Perseus [1] proteins were hierarchically clustered using Euclidian Distance as matrix (log transformed SILAC protein ratios). To address the biological implications of the proteins in

each cluster, Gene Ontology terms were retrieved and tested for enrichment compared to the remainder of the dataset using the Fishers' exact test ($p<0.05$).

H_2O_2 assay

H_2O_2 levels were analyzed by the HRP/H_2O_2 kit from Abcam according to manufacturer`s instructions, briefly: fluorescence (ex530/em580nm) was measured and normalized to non-treated cells and to cell count. The fold change in fluorescence in emodin-treated cells was depicted.

BrdU assay

3 x 10^4 NHF cells or 5 x 10^4 cells of each cancer lines were seeded pro well of a 24 well plate, containing glass cover slides coated with collagen I. Two biological replicates were done for each cell under each treatment.

After cells become adherent, medium was removed and cells were washed twice with DPBS, and let overnight in medium free of FCS in order to synchronize their replicative cycle. Overnight medium was changed to normal medium including control or 70 uM emodin treatment for 24 hours

Cells were supplemented with 3 µM BrdU (Sigma B5002) for 4 hours (incubated at 37°C and 5% CO2), then thoroughly washed wirh DPBS and fixed with 50% methanol, 50% acetone (2 minutes at room temperature). Cells were blocked with 3 % BSA in DPBS for 15 min at room temperature. For cell permeabilization and DNA-denaturation 0,5 % Triton X-100 in 2 M HCl was used for 10 minutes at room temperature.

Cells were incubated overnight at 4°C in blocking solution containing BrdU antibody (Dako, mouse, 1:50) and RNase (Sigma, 1:1000). Secondary antibody Alexa 488 donkey anti-mouse (Invitrogen A21202) in a ratio 1:2000 was used for one hour at room temperature. Coverslips were mounted with ProLong Gold mounting medium.

Yeast strains and growth conditions

Yeast strains were streaked out on rich medium agar plates (YPD), inoculated in rich medium liquid media (YDP), and grown over night in quadruple-indented flasks at 30°C with 140 rpm. Cultures were inoculated to an optical density (OD600) of 0.1 in synthetic complete media (SC), according to Sherman [3], with glucose (Glc) (2%) or galactose/lactate (GalLac) (2%, 0.5%) as carbon sources. Cultures were treated with 50 µM emodin (dissolved in ethanol) or with ethanol, respectively. For determining the role of oxidative stress in emodin-triggered cytotoxicity, yeast cells were simultaneously treated with increasing concentrations of N-acetyl cysteine (NAC) (freshly prepared in ddH2O).

Yeast strain	Strain type	Genetic background	References
BYa wt	BY4741	MATa, *his3*Δ1; *leu2*Δ0; *met15*Δ0; *ura3*Δ0; ρ^+	EUROSCARF
BYa wt ρ^0	BY4741	MATa, *his3*Δ1;*leu2*Δ0; *met15*Δ0; *ura3*Δ0; ρ^0	[2]

Measurement of yeast cell survival

Yeast cell survival is determined by a clonogenic assay, which measures the ability of treated or untreated yeast cells to from new colonies upon optimal growth conditions (rich medium agar plates with glucose as the sole carbon source, YPD) [4]. Cell densities of treated or untreated yeast cultures were measured with an automated cell counter (Z2 Coulter Particle Count and Size Analyzer, Beckman Coulter, Krefeld, Germany; equipped with a 50 µm aperture; counted particles were in the size of 2 to 6.7 µm; each sample was measured in duplicate). 500 cells of each condition were plated on YPD at least in duplicate. The colony forming units (CFUs), i.e., the number of colonies grown after two days of incubation at 28°C were determined.

Measurement of oxidative stress in yeast

Oxidative stress was determined by measuring the conversion of dihydroethidium (DHE, Sigma-Aldrich, Taufkirchen, Germany) to the red fluorescent ethidium applying a fluorescence plate reader [5]. 30 µL per sample were pelleted in 96-well plates. Cell pellets in each well were resuspended in 200 µL DHE-staining solution (2.5 µg/mL in PBS for DHE; 2.5 mg/mL DHE stock solution in DMSO). After 10 min of incubation at RT, OD600 and the ethidium fluorescence were measured in a FLUOstar Omega fluorescence plate reader (BMG Labtech, Ortenberg, Germany) with the following settings for OD600: mode well scanning, number of flashes per scan point 5, well scanning 5x5, diameter 2 mm, and for fluorescence: mode endpoint, fluorescence top, excitation 544 nm, emission 620 nm, gain 2000, number of flashes per well 10, orbital averaging 2 mm. The ratio of fluorescence intensity (relative fluorescence units, RFUs) to cell mass (OD600) was used as measurement of ROS accumulation in the different samples. Staining solution was used for blank measurements. Samples were measured at least in quadruplicate.

Measurement of oxygen consumption

A Clark type polarographic oxygen sensor (Oxygraph 2-k, Oroboros Instruments) was used to measure oxygen consumption. For measurements with isolated yeast mitochondria 2 ml of respiratory buffer (0.6 M sorbitol, 10 mM MgCl2, 0.5 mM EDTA, 20 mM potassium phosphate, pH 7.2) were pre-warmed in a temperate chamber with a close-fitting lid. Mitochondria were added (100 µg total mitochondrial protein) and measurements were carried out at 30°C. Substrate (5 mM succinate), ADP (300 µM), oligomycin (500 nM), valinomycin (10 nM) or emodin (various indicated concentrations) were added at the given time points. For measurements with human cell lines cells were trypsinized and resuspended in pre-warmed DMEM (high Glucose). Measurements were carried out at 37°C. After a stable baseline was reached either CCCP or emodin was added at the indicated concentrations.

In vitro protein synthesis and import into isolated mitochondria

Radiolabeled Su9-DHFR was synthesized as described [6]. Import of the [^{35}S]Su9-DHFR precursor protein was carried out in import buffer (0.7% [w/v] bovine serum albumin, 10 mM MOPS, pH 7.2, 250 mM sucrose, 80 mM KCl, 5 mM MgCl2, 2 mM KH2PO4, 5 mM methionine, 4 mM ATP, 4 mM NADH, 10 mM creatine phosphate and 0.2 mg/ml creatine kinase) at 25°C for 10 minutes. Prior to import either indicated concentrations of emodin or ethanol were added to the import mix. Import reactions were stopped by the addition of an AVO mix (8 µM antimycin A, 1 µM valinomycin, 20 µM oligomycin), mitochondria were re-isolated and non-imported precursor proteins were removed by washing with SEM buffer (250 mM sucrose, 1 mM EDTA, 10 mM MOPS [pH 7.2]). Samples were analyzed by SDS-PAGE and autoradiography.

Identification of emodin binding partners

Emodin was resuspended in 50% N,N-Dimethylformamid (DMF), pH 13 immobilized on Epoxy-Activated Sepharose 6B beads (GE health care, Freiburg, Germany) according to manufacturer's instructions. Sepharose 6B beads were suspended in distilled and washed with 200 ml distilled water for one hour. Bead slurry and emodin solution were mixed in a 1:1 ratio and incubated in a thermo mixer for 16 h at 40°C/800 rpm. Excess emodin was washed away with 50 % DMF, pH 13 and free binding sites were blocked by incubation with 1 M ethanolamine, pH 8 overnight at 40 °C. Coupled beads were washed with three cycles of 0.1 NaOAc /0.5 M NaCl/ pH 4 and 0.1 M Tris-HCl/0.5 M NaCl/pH 8. Control beads were treated as described, without coupling of emodin.

SILAC labeled cell pellets of three 15 cm-cell culture dishes were homogenized in 3 volumes of modified RIPA buffer (1% NP-40, 150 mM NaCl, 50 mM Tris, pH 7.5, 0,25% sodium deoxycholate and freshly added protease inhibitors (Roche, Basel, Switzerland) by use of a vortex mixer. Differentially labeled lysates were incubated with emodin beads or control beads by end-over-end rotation overnight at 4°C. Beads were washed three times with modified RIPA buffer and bound proteins were eluted by boiling at 95°C for 10 min in 2x SDS loading buffer (0.3 M Tris, 12% SDS, 40% glycerol, 0.05% bromphenolblue), and reduced and alkylated by 1 mM dithiotreitol and 5.5 mM iodoacetamide.

Heavy and light labeled eluates were mixed in a 1:1 ratio and separated in a 4-12% NuPAGE gel (Invitrogen, Darmstadt, Germany). An in-gel tryptic digestion and STAGE tip purifications were performed as described.

REFERENCES

1. J. Cox, M. Mann, MaxQuant enables high peptide identification rates, individualized p.p.b.-range mass accuracies and proteome-wide protein quantification, Nat Biotechnol, 26 (2008) 1367-1372.
2. S. Buttner, A. Bitto, J. Ring, M. Augsten, P. Zabrocki, T. Eisenberg, H. Jungwirth, S. Hutter, D. Carmona-Gutierrez, G. Kroemer, J. Winderickx, F. Madeo, Functional mitochondria are required for alpha-synuclein toxicity in aging yeast, J Biol Chem, 283 (2008) 7554-7560.
3. F. Sherman, Getting started with yeast, Methods Enzymol, 350 (2002) 3-41.
4. R.J. Braun, C. Sommer, D. Carmona-Gutierrez, C.M. Khoury, J. Ring, S. Buttner, F. Madeo, Neurotoxic 43-kDa TAR DNA-binding protein (TDP-43) triggers mitochondrion-dependent programmed cell death in yeast, J Biol Chem, 286 (2011) 19958-19972.
5. R.J. Braun, C. Sommer, C. Leibiger, R.J. Gentier, V.I. Dumit, K. Paduch, T. Eisenberg, L. Habernig, G. Trausinger, C. Magnes, T. Pieber, F. Sinner, J. Dengjel, F.W. van Leeuwen, G. Kroemer, F. Madeo, Accumulation of Basic Amino Acids at Mitochondria Dictates the Cytotoxicity of Aberrant Ubiquitin, Cell Rep, (2015).
6. D. Stojanovski, N. Pfanner, N. Wiedemann, Import of proteins into mitochondria, Methods Cell Biol, 80 (2007) 783-806.

SUPPLEMENTARY FIGURES AND TABLES

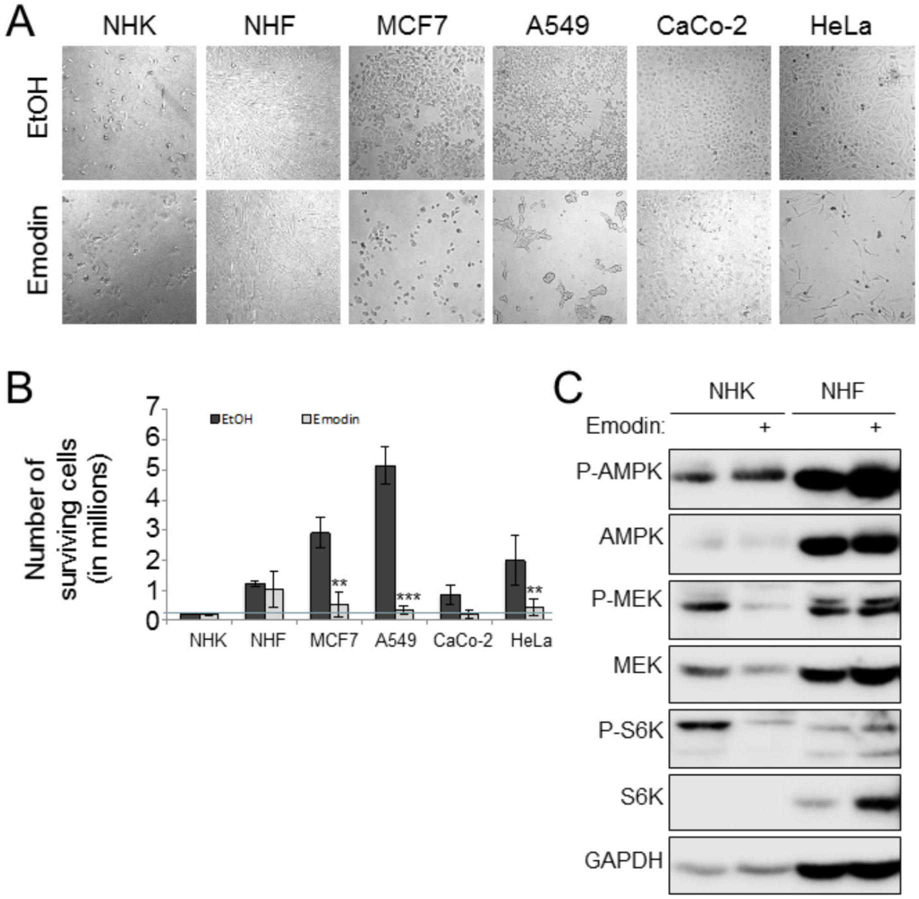

Supplementary Figure 1: Effect of emodin on healthy and cancer cells. Evaluated cells are normal human fibroblasts (NHF), normal human keratinocytes (NHK) and four cancer cell lines: MCF7, A549, CaCo-2 and HeLa. **(A)** Phenotype of cells treated with 70 µM emodin after 48 h compared to control treatment with ethanol (EtOH). **(B)** Relative number of cells after two days of treatment. Initial number of cells is 150,000 and is highlighted by a light blue line. Shown are average numbers of biological triplicates. Error bars indicate standard deviations. T-test;**: $p < 0.01$; ***: $p < 0.001$. **(C)** Western blots of phospho-ribosomal protein S6 kinase (P-S6K, Thr389) phospho-AMPKα (P-AMPK, Thr172) and phospho-MEK (P-MEK, Ser217/221). Western blots against respective non-modified proteins and GAPDH were used as loading control.

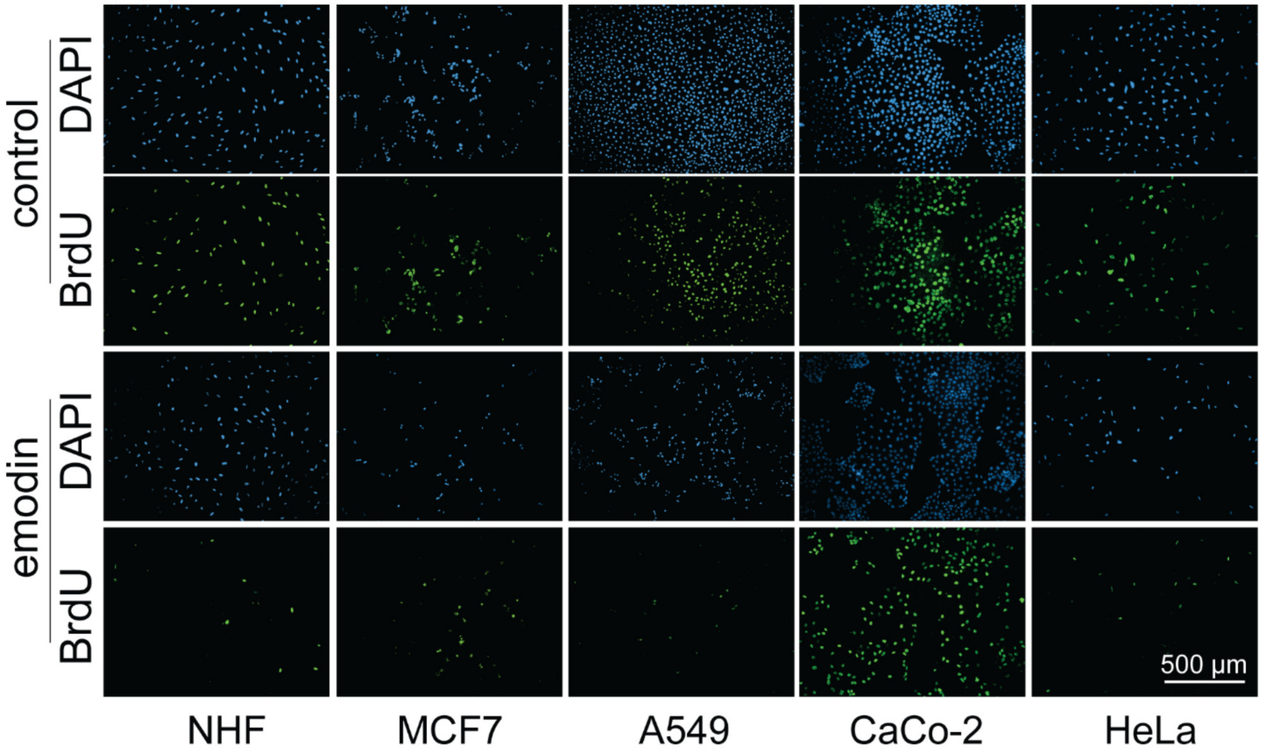

Supplementary Figure 2: BrdU assay of cells treated for 24 h with 70 µM emodin compared to control conditions to determine percentage of proliferating cells.

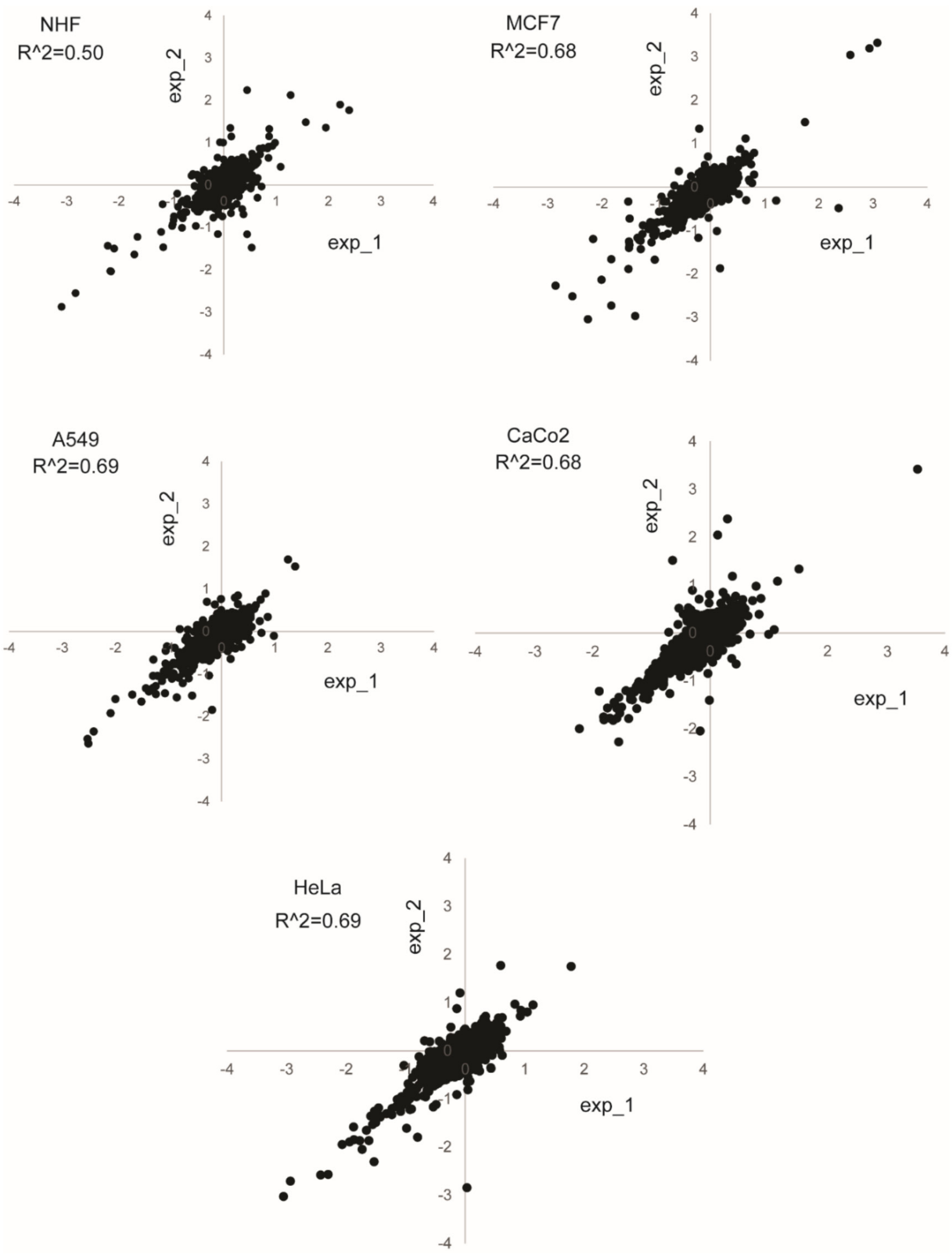

Supplementary Figure 3: Correlation between biological replicates of proteomics experiments (log_2 SILAC ratios). Different SILAC labels were used for emodin and control treatments.

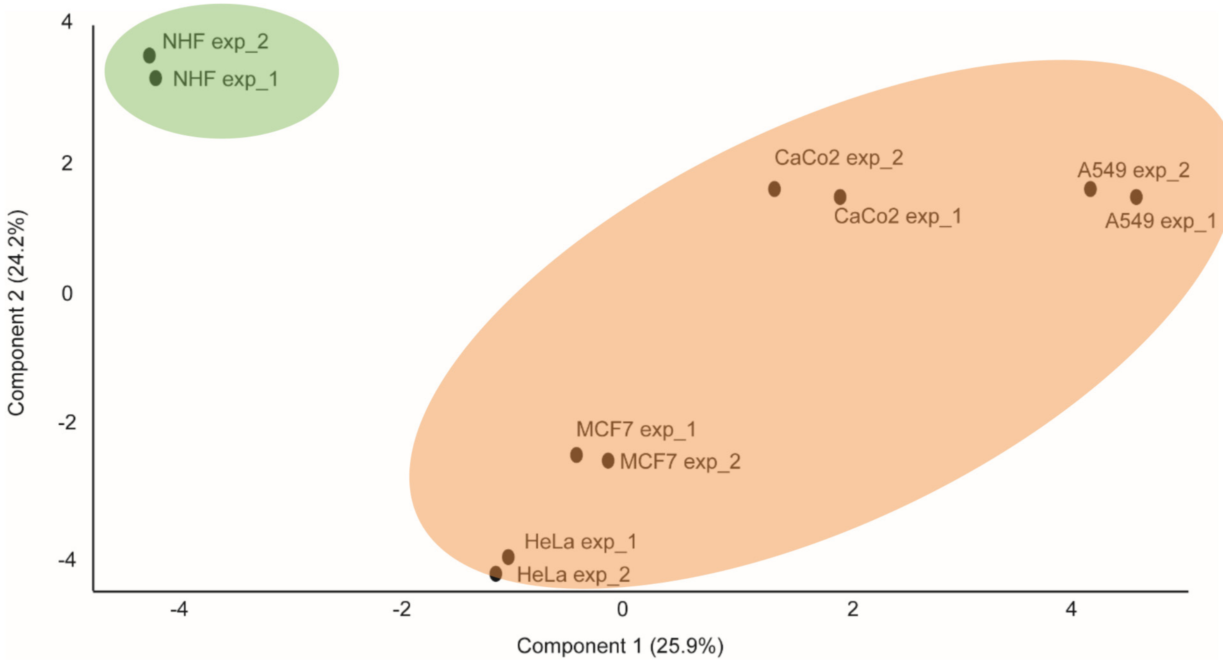

Supplementary Figure 4: Log_2-transformed abundance changes of proteins under emodin treatment compared to control conditions were used for a principal component analysis. Healthy cells treated with emodin (NHF) are clearly separated from cancer cells under the same conditions.

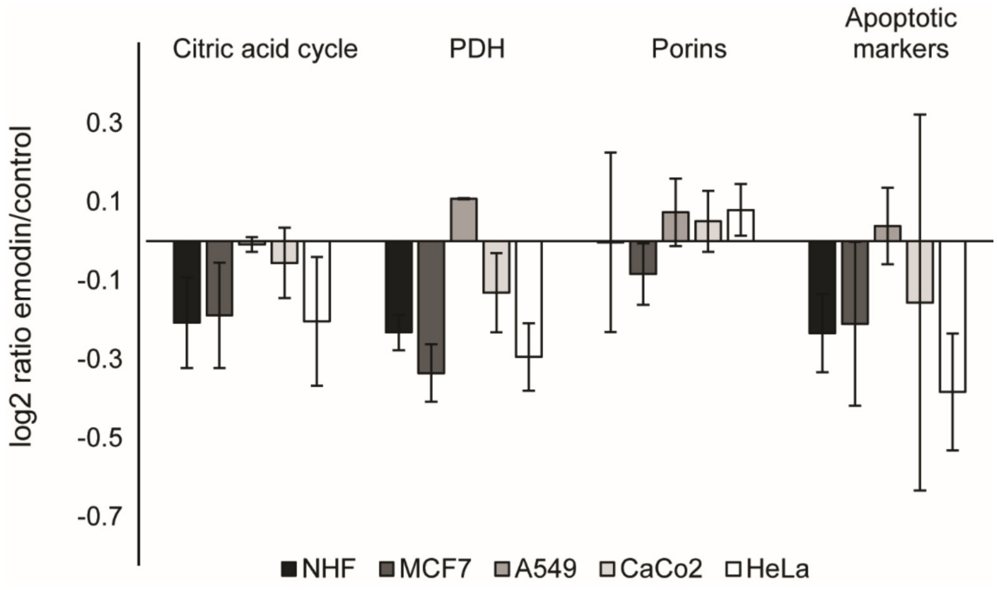

Supplementary Figure 5: Log_2-transformed levels of different mitochondrial protein groups in the different cells under emodin treatment show no significant changes compared to control conditions. Error bars: standard deviation.

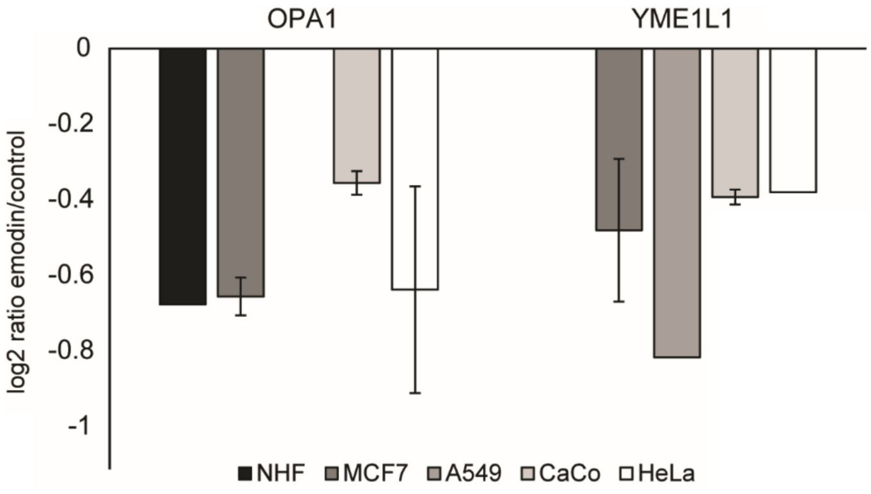

Supplementary Figure 6: Log_2-transformed levels of the mitochondrial fusion protein OPA1 and of the protease YME1L1 proteins known to be involved mitochondrial fragmentation upon emodin treatment. Note that not all evaluated proteins were detected in all cells.

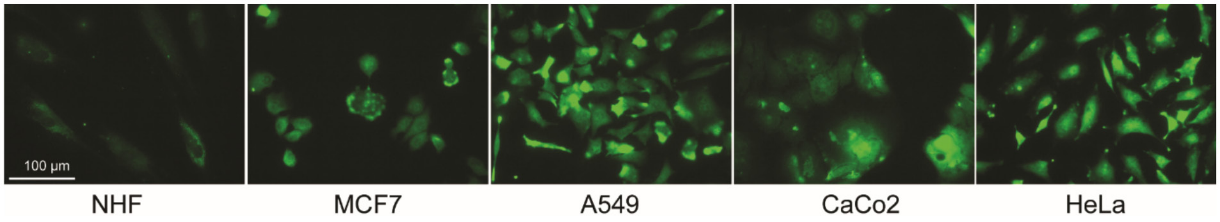

Supplementary Figure 7: Inherent ROS levels in evaluated cells under control conditions as revealed by fluorescent assay (CellROX Green Reagent). ROS levels are higher in cancer than in healthy cells.

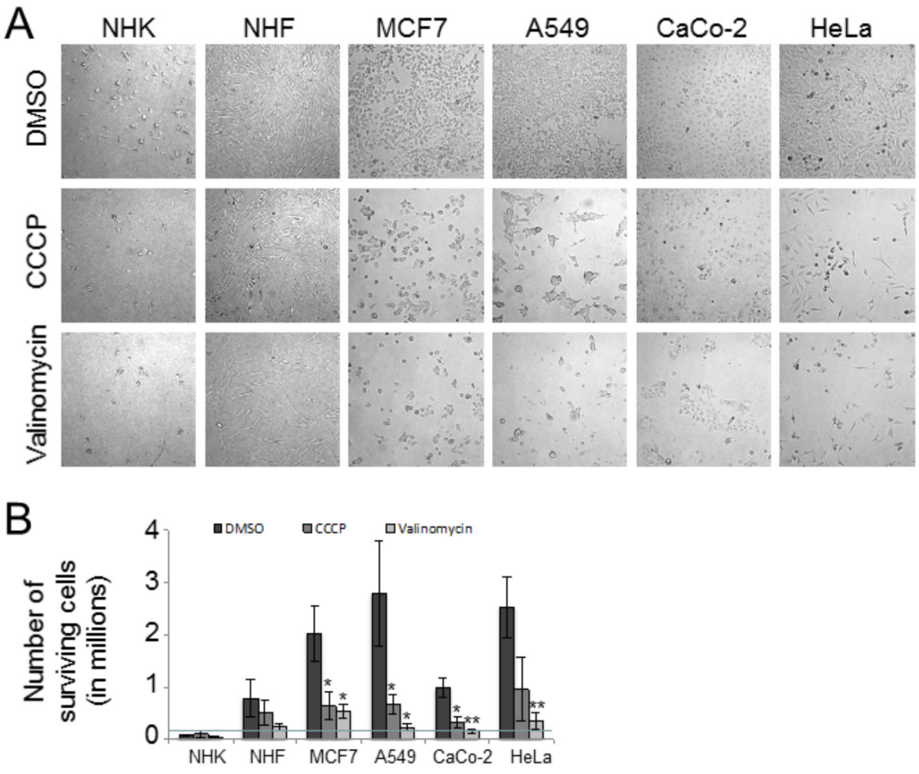

Supplementary Figure 8: Effect of CCCP and valinomycin on healthy and cancer cells. Evaluated cells are normal human fibroblasts (NHF), normal human keratinocytes (NHK) and four cancer cell lines: MCF7, A549, CaCo-2 and HeLa. **(A)** Phenotype of cells treated with 10 µM CCCP or 10 µM valinomycin after 48 h compared to control treatment with DMSO. **(B)** Relative number of cells after two days of treatment. Initial number of cells is 150,000 and is highlighted by a light blue line. Shown are average numbers of biological triplicates. Error bars indicate standard deviations. T-test; *: $p < 0.05$; **: $p < 0.01$.

Supplementary Table 1: List of detected proteins and their log_2 values (emodin to control ratio) in emodin affinity purifications as detected by LC-MS/MS in three biological replicates.

See Supplementary File 1

Supplementary Table 2: List of detected proteins and their log_2 values (emodin to control ratio) as detected by LC-MS/MS for each cell type measured in two biological replicates.

See Supplementary File 2

Supplementary Table 3: List of proteins detected for the five different evaluated cell types and the average of detected ratios for each cell type (emodin to control ratio, and log_2-transformed) considering results from two biological replicates. Proteins which proved to be significantly changed are marked by a + sign.

See Supplementary File 3
